# The timing of durvalumab administration affects the risk of pneumonitis in patients with locally advanced non-small cell lung cancer: a systematic review and meta-analysis

**DOI:** 10.1186/s12885-023-11472-3

**Published:** 2023-10-10

**Authors:** Zhenyi Yang, Wen Zhong, Yixuan Luo, Chunli Wu

**Affiliations:** The Fourth Affiliated Hospital of China Medical University, Chongshan East Road #4, Huanggu District, Liaoning, 110032 China

**Keywords:** Non-small cell lung cancer, Radiotherapy, Durvalumab, Time interval, meta- analysis

## Abstract

**Purpose:**

The PACIFIC study has demonstrated that the administration of durvalumab following concurrent chemoradiotherapy can significantly improve both overall survival and progression-free survival rates in patients with locally advanced unresectable non-small cell lung cancer. While the latest NCCN guidelines recommend this combination regimen, they do not specify the optimal timing for administering durvalumab after completing radiotherapy. The PACIFIC study suggested initiating durvalumab within 42 days of completing radiotherapy, but early administration of the drug may increase the incidence of pneumonitis. Therefore, we conducted this study to investigate whether the time interval between completion of radiotherapy and initiation of durvalumab treatment is associated with the risk of pneumonitis (Grade ≥ 3), which is the primary endpoint, as well as progression-free survival, which is the secondary endpoint.

**Methods:**

A comprehensive search of clinical trials in PubMed and EMBASE was conducted up to March 2023 to identify clinical trials involving locally advanced unresectable non-small cell lung cancer patients who were treated with durvalumab following chemoradiotherapy. Meta-analysis was performed on single-arm studies to estimate the incidence of pneumonitis (Grade ≥ 3) and progression-free survival in all studies, as well as in studies that administered durvalumab within 42 days after completion of radiotherapy.

**Results:**

This meta-analysis consisted of nine studies with a total of 2560 patients. The analysis showed that the incidence of pneumonitis (Grade ≥ 3) was 5.36% [95%CI (0.03, 0.08), I^2^ = 18.41%, *p* = 0.29], while the 1-year progression-free survival rate was 57.91% [95%CI (0.53, 0.63), I^2^ = 10.57%, *p* = 0.35]. Furthermore, when the duration between completion of radiotherapy and initiation of durvalumab treatment was shorter than 42 days, the incidence of pneumonitis (Grade ≥ 3) was 4.12% [95%CI (0.02, 0.06), I^2^ = 0.00%, *p* = 0.56], with a 1-year progression-free survival rate of 61.03% [95%CI (0.51, 0.71), I^2^ = 59.06%, *p* = 0.09].

**Conclusion:**

Overall, based on the available evidence, it appears that there is no significant increase in pneumonitis or decrease in progression-free survival (PFS) when the time interval is less than 42 days and a shorter interval between treatment sessions does not necessarily have a detrimental effect on the rate of pneumonitis. We recommend that clinicians carefully evaluate the specific circumstances of each patient to determine the optimal timing for initiating immunotherapy.

## Introduction

Overall, lung cancer remains one of the most dangerous malignancies in the world and is the leading cause of cancer-related death. Non-small cell lung cancer (NSCLC), a subtype of lung cancer, accounts for the majority of cases, with surgery being the primary treatment for locally advanced NSCLC [1]. However, due to factors such as tumor size and overall health status, surgery is often not possible. For patients with unresectable locally advanced NSCLC, concurrent chemoradiotherapy is standard treatment. Recent clinical studies, such as PACIFIC, suggest that immunotherapy with programmed cell death-1 (PD-1) receptor or programmed cell death-ligand 1 (PD-L1) inhibitors following chemoradiotherapy can significantly enhance patient outcomes in terms of overall survival (OS) and progression-free survival (PFS) [2–5]. This is especially applicable to patients with inoperable, locally advanced NSCLC. In the PACIFIC study, locally advanced NSCLC patients who received chemoradiotherapy and PD-L1 immune checkpoint inhibitor durvalumab had significantly longer OS and PFS than those treated with chemoradiotherapy alone [6]. The latest NCCN guidelines recommend incorporating durvalumab into NSCLC management after radiotherapy and chemotherapy completion, but there is no consensus on optimum timing for administering durvalumab after radiation therapy. As per the PACIFIC study, durvalumab treatment initiated up to 42 days after the final radiation session, and the greatest benefit is obtained when treatment commences between 1- and 14-days following radiation [6].

Radiation therapy can provide a robust immunomodulatory effect, creating a supportive immune microenvironment for anti-tumor immunity. Studies have revealed that radiation-induced anti-tumor immunity efficacy may depend on dose; higher doses demonstrate a stronger immune adjuvant effect [7, 8]. Indeed, radiation therapy offers several immunomodulatory effects such as increasing antigen presentation, releasing chemokines, attracting effector T-cells to the tumor microenvironment, and promoting immunogenic cell death mediated by lymphocytes [9]. Activation of negative T-cell regulation pathways like PD-1/PD-L1 axis following radiation may contribute to these effects [10]. It is crucial to note that radiation therapy can upregulate the expression of PD-1 and PD-L1 on immune and tumor cells, making its combined use with PD-1/PD-L1 inhibitors particularly relevant [10–12].

Incorporating a new treatment into standard cancer therapy is crucial, but balancing efficacy and safety is equally important. Immunotherapy with PD-1/PD-L1 inhibitors may affect other organ systems, and lead to immune-related pneumonitis, which poses the most significant potential fatalities among all reported adverse events [13–15]. The incidence of all grades of immune-related pneumonitis in clinical trial data ranges from 2 to 38%, while the incidence of Grade ≥ 3 immune-related pneumonitis ranges from 0.6 to 2.7% [16]. Additionally, based on real-world data, the incidence of immune-related pneumonitis in non-small cell lung cancer patients varies from 4.8 to 39.3%. Radiation therapy itself can also cause dose and radiation volume-dependent radiation pneumonitis in some patients [17]. In thoracic radiotherapy patients, the incidence of Grade 3 or higher radiation pneumonitis is between 1.8 and 10.0% [18–22]. Recently, a meta-analysis found that combining radiotherapy and chemotherapy with PD-1/PD-L1 inhibitors increases the incidence of Grades 1–2 immune-related pneumonitis or radiation pneumonitis but does not increase Grade ≥ 3 pneumonitis incidence [23]. Thus, PD-1/PD-L1 inhibitors offer excellent efficacy and good safety. The PACIFIC study initiated durvalumab treatment between 1 and 42 days after the final radiation therapy session. The latest NCCN guidelines recommend administering durvalumab treatment after the completion of radiotherapy and chemotherapy, but they do not specify when the therapy should be started after the final radiation therapy session. This study aims to investigate whether the time interval between the final radiation therapy session and durvalumab treatment is associated with the risk of pneumonitis, with the primary study endpoint being the incidence of Grade ≥ 3 pneumonitis. We believe that assessing the risk of pneumonitis alone is insufficient without considering survival. Hence, our original intention was to examine whether the timing of durvalumab treatment after chemoradiotherapy would impact survival, including overall survival and progression-free survival. However, due to the significant lack of overall survival data, we can only consider progression-free survival as a secondary endpoint for now.

Immunotherapy-related pneumonitis and radiation pneumonitis can be challenging to distinguish clinically. Many studies do not differentiate between the two when reporting adverse reactions. In addition to these two types of pneumonitis, patients may develop other forms of pneumonitis both during treatment and during the follow-up period. Whether it is immune-related pneumonitis, radiation pneumonitis, or other types of pneumonias that occur during treatment, they all have a significant impact on the treatment and prognosis of cancer patients. It should be noted that many studies do not specifically categorize pneumonitis cases. Therefore, this study aims to encompass various types of pneumonitis that can occur during treatment. This includes immune-related pneumonitis, radiation pneumonitis, and other forms of pneumonitis. By including these different types of pneumonitis, the study aims to provide a comprehensive assessment of the overall burden on cancer patients undergoing treatment. We also evaluate other factors that influence the risk of pneumonitis, such as radiation doses and populations. While many other factors can also impact the risk of pneumonitis, given the lack of extensive research data, this study still holds significant value and can contribute to optimizing combined treatment strategies.

## Methods

### Literature search

This systematic review and meta-analysis followed the Preferred Reporting Items for Systematic Reviews and Meta-Analyses (PRISMA) guidelines [24–26] and has been registered on the PROSPERO database (CRD42023423736). A comprehensive search was conducted up to March 2023 in PubMed, Cochrane Library, and EMBASE using the algorithm “Non-Small Cell Lung cancer,” “Radiotherapy,” “Durvalumab” by Mr. Yang and Mrs. Zhong. Any conflicts and uncertainties were resolved by Mrs. Wu.

The inclusion criteria for this study were: (1) tumor: unresectable locally advanced non-small cell lung cancer; (2) treatment: chemoradiotherapy flowed by durvalumab; (3) time interval: the time interval between durvalumab and chemoradiotherapy was reported; (4) results: the incidence of pneumonitis (Grade ≥ 3) was reported.

The exclusion criteria for this study were: (1) tumor: other malignancies; (2) tumor progression: local recurrence or metastasis before durvalumab; (3) results: data could not be extracted, or the incidence of pneumonitis was 0%; (4) text: reviews, editorials, dispatches, protocols; (5) version: repeated reports (only the latest data was included).

### The risk of bias

The assessment of bias risk was performed by Mr. Yang and Mrs. Zhong using the Cochrane Risk of Bias tool or MINORS. All trials were graded as high risk, unclear risk, or low risk. Data extraction was performed by Mr. Yang and Mrs. Zhong and validated independently by Mrs. Wu.

### Statistical analysis

This meta-analysis was conducted using Stata 14. The incidence of pneumonitis (Grade ≥ 3) was analyzed using binary analysis. The heterogeneity between comparisons was estimated using the I-squared statistic.

## Results

### Characteristics of all trials

**Fig. 1:**
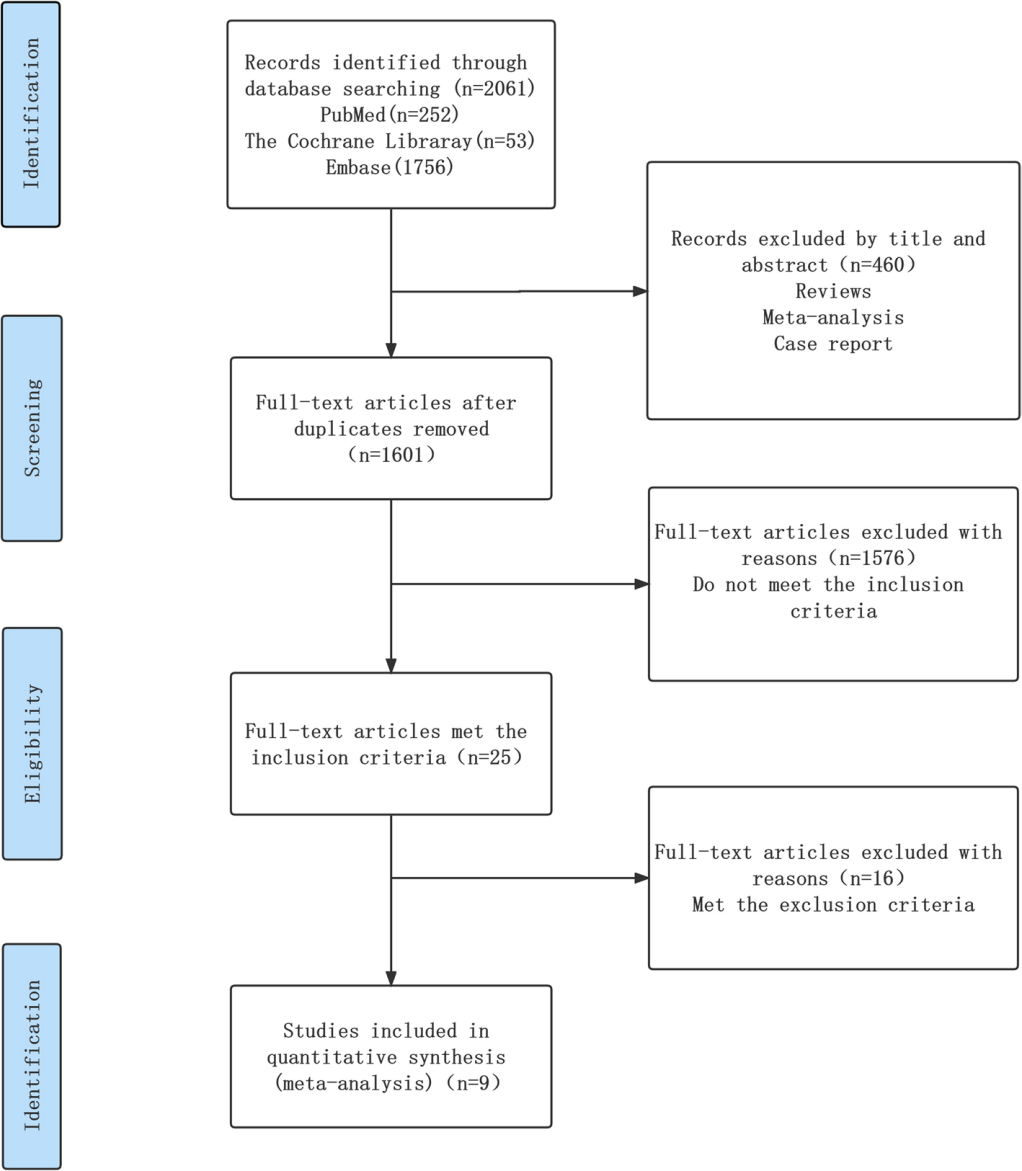
Flow diagram

 This meta-analysis included a total of 9 studies and 2560 patients [27–35]. All patients had completed chemoradiotherapy prior to receiving durvalumab, and no local recurrence or metastasis was present before durvalumab administration. Eight of the studies were retrospective single-arm trials [28–35], while one was a randomized controlled trial [27]. The time interval between completion of radiotherapy and durvalumab administration was reported in 3 studies to be within 1–42 days [27, 32, 33]. The time interval was reported as 0-157 days [28], 1.8–3.7 months [29], 35–981 days [30], 10–84 days [31], ≥ 14 days [34], and 13–103 days [35] in the remaining 6 studies. The study conducted by Girard et al. was indeed excluded from the analysis due to the wide range of interval durations, ranging from 35 to 981 days [30]. This considerable variation raised concerns about whether the 981-day period could still be considered as consolidation therapy. To ensure the credibility of the results, the decision was made to focus on studies with a more consistent and well-defined duration for consolidation therapy. By doing so, the study aimed to improve the reliability and accuracy of the findings. Harada conducted a subgroup analysis, where the time interval was reported as 10–14 days and 14–84 days [31]. The number of patients with pneumonitis (Grade ≥ 3) was reported as 3 and 0, respectively. Pneumonitis grading was evaluated according to the National Cancer Institute Standard Common Terminology for Adverse Events Pneumonia grading criteria. Figure 1 shows the flow diagram, and Table 1 summarizes the basic characteristics of all studies.

**Table 1:** Characteristics of included studies

Author	Year	Nation	Study type	Patients (n)	Tumor stage	Radiation therapy technique	Radiation dose (Gy	Immune therapy	Time interval	Incidence of pneumonitis (grade ≥3)	1-year PFS
Ares *et al.*	2020	Multicenter	RCT	475	III	None	54-74	Durvalumab	1-42 days	4.63%	55.90%
Avrillon *et al.*	2022	France	Retrospective trial	454	III	None	45-74	Durvalumab	0-157 days	1.32%	None
Chu *et al.*	2023	China	Retrospective trial	31	III	None	60-70	Durvalumab	1.8-3.7 months	6.90%	56.40%
Girard *et al.*	2022	Multicenter	Retrospective trial	1399	III	None	60-66	Durvalumab	35-981 days	3.29%	62.20%
Harada *et al.*	2022	Japan	Retrospective trial	26	III	IMRT	60	Durvalumab	10-84 days	11.54%	51.80%
Huang *et al.*	2022	Singapore	Retrospective trial	39	III	IMRT VMAT	60-66	Durvalumab	1-42 days	7.69%	59.60%
Miura *et al.*	2020	Japan	Retrospective trial	41	III	IGRT	54/60	Durvalumab	1-42 days	2.44%	73.17%
Saad *et al.*	2022	Israel	Retrospective trial	71	III	IMRT VMAT	56-66	Durvalumab	≥14 days	5.63%	83.10%
Taugner *et al.*	2021	Germany	Retrospective trial	26	III	None	≥60	Durvalumab	13-103 days	15.38%	62.00%

### The risk of bias

The risk of bias in the 8 studies included in the meta-analysis was assessed by Mr. Yang and Mrs. Zhong. They utilized the Cochrane Risk of Bias tool or the Methodological Index for Non-Randomized Studies (MINORS) to evaluate and determine the risk of detection, reporting, and attrition bias in each study. The assessment conducted by Mr. Yang and Mrs. Zhong concluded that all the included studies demonstrated a low risk of bias in these aspects. This rigorous evaluation process enhances the reliability and credibility of the meta-analysis results by ensuring that the included studies have minimized potential biases in their methodology and reporting.

### Outcomes

**Fig. 2:**
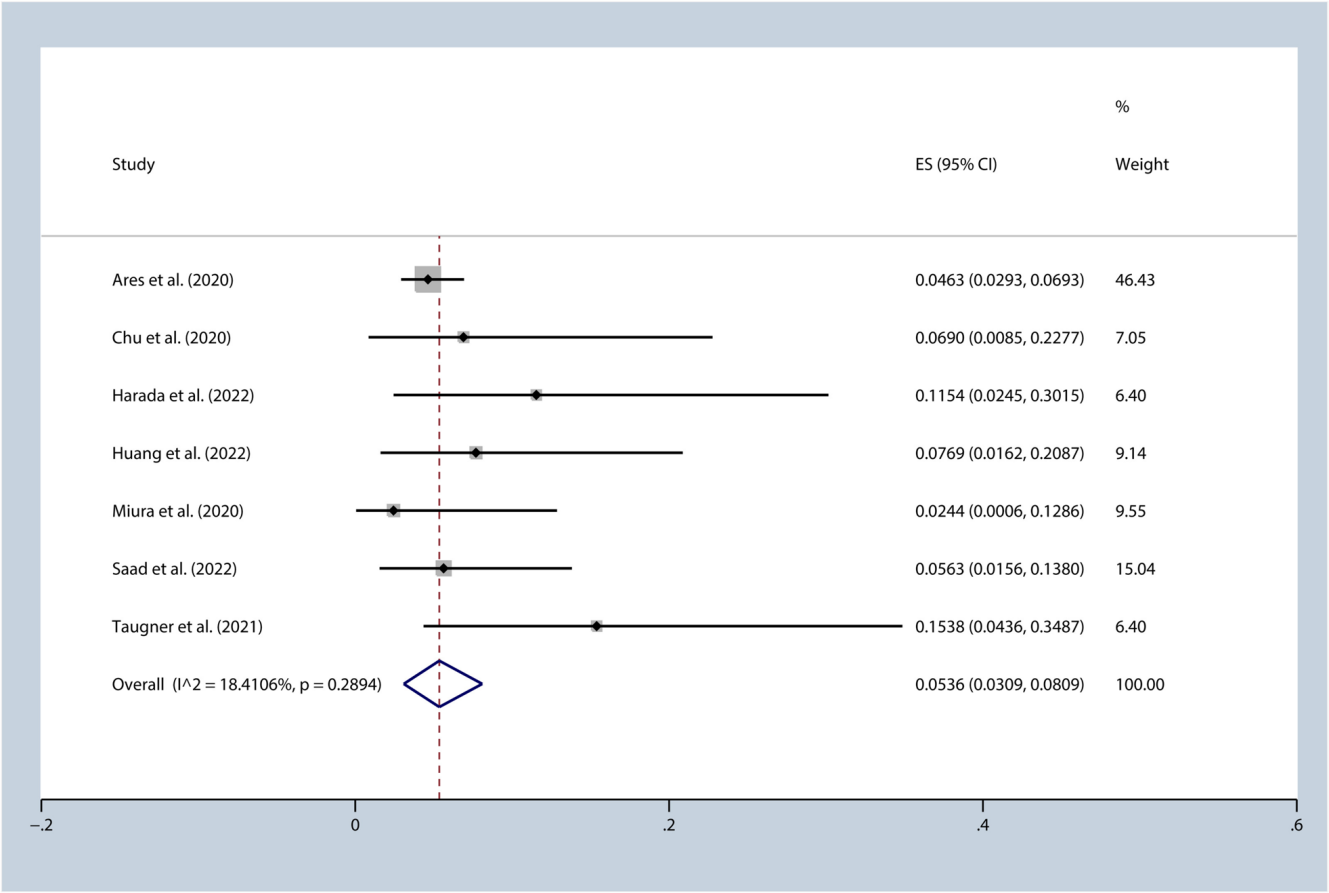
Incidence of pneumonitis (≥grade 3) (overall analysis)

**Fig. 3:**
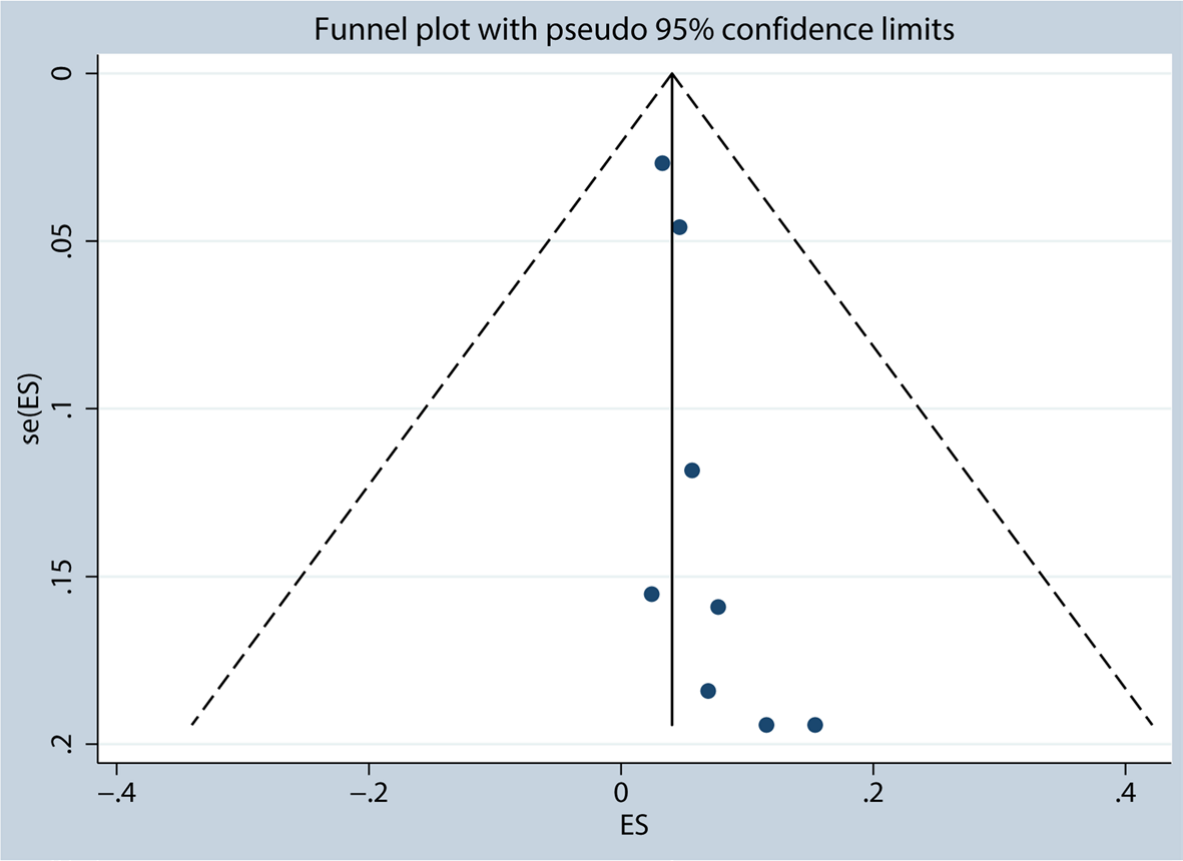
Funnel plot

 A heterogeneity test was conducted on a set of 8 studies which showed that the I^2^ value was 70.46%, indicating moderate heterogeneity. A sensitivity analysis was performed where each study was excluded one-by-one, revealing that study Avrillon et al. was the source of the heterogeneity [28]. After removing this study, the I^2^ value dropped significantly, which suggests that there was a reduced level of heterogeneity. The incidence of pneumonitis (Grade ≥ 3) was 5.36% [95%CI (0.03, 0.08), I^2^ = 18.41%, *p* = 0.29], as shown in Fig. 2. To evaluate publication bias, a funnel plot and Egger’s test were used. The funnel plot, as depicted in Fig. 3, was used to assess publication bias in the meta-analysis. Additionally, Egger’s test was conducted, and the results indicated no significant publication bias, as evidenced by a *p*-value of 0.11. This suggests that there was no substantial asymmetry in the distribution of study effect sizes, indicating that the likelihood of publication bias influencing the results of the meta-analysis is minimal. These findings contribute to the overall validity and robustness of the study’s conclusions, as they suggest that the included studies were representative and not unduly influenced by publication bias. Furthermore, the trim-and-fill method demonstrated that there was no bias risk, indicating that the results were stable. Among the total of 7 studies that were analyzed for 1-year PFS rate, a heterogeneity test revealed I^2^ = 77.34%, indicating high heterogeneity. Upon sensitivity analysis, it was discovered that study Saad et al. was responsible for the heterogeneity [34]. After its removal, the I^2^ value dropped significantly, which suggests reduced heterogeneity and a 1-year PFS rate of 57.91% [95%CI (0.53, 0.63), I^2^ = 10.57%, *p* = 0.35].

**Fig. 4:**
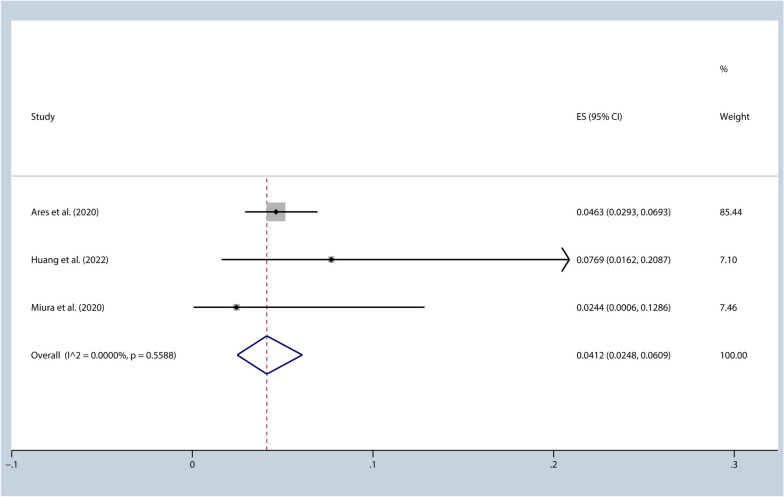
Incidence of pneumonitis (≥grade 3) (time interval ≤42days)

 A meta-analysis was conducted on 3 studies with time intervals ranging from 1 to 42 days, as well as a subgroup from Harada et al. with intervals less than 14 days [27, 31–33]. The results showed an I^2^ value of 67.77%, indicating moderate heterogeneity. A sensitivity analysis was performed, and it was found that Harada et al. was the source of heterogeneity [31]. Upon exclusion of this study, the I^2^ = 0.00%, while the incidence of pneumonitis (Grade ≥ 3) was 4.12% [95%CI (0.02, 0.06), I^2^ = 0.00%, *p* = 0.56] (Fig. 4) and the 1-year PFS rate was 61.03% [95%CI (0.51, 0.71), I^2^ = 59.06%, *p* = 0.09]. Among the studies with intervals greater than 42 days were Chu et al. and Saad et al. [29, 34]. For Chu et al., the incidence of grade 3 or higher pneumonitis and the 1-year PFS rate were 5.63% and 56.40% respectively. For Saad et al., the incidence of pneumonia (Grade ≥ 3) was 6.90%. Harada et al. showed that a shorter interval between radiotherapy and Durvalumab treatment resulted in a higher incidence of grade 3 or higher pneumonitis when compared to an interval greater than 14 days [31]. Unfortunately, since the only subgroup in the 9 studies with an interval less than 14 days was from study Harada et al., further analysis could not be performed.

**Fig. 5:**
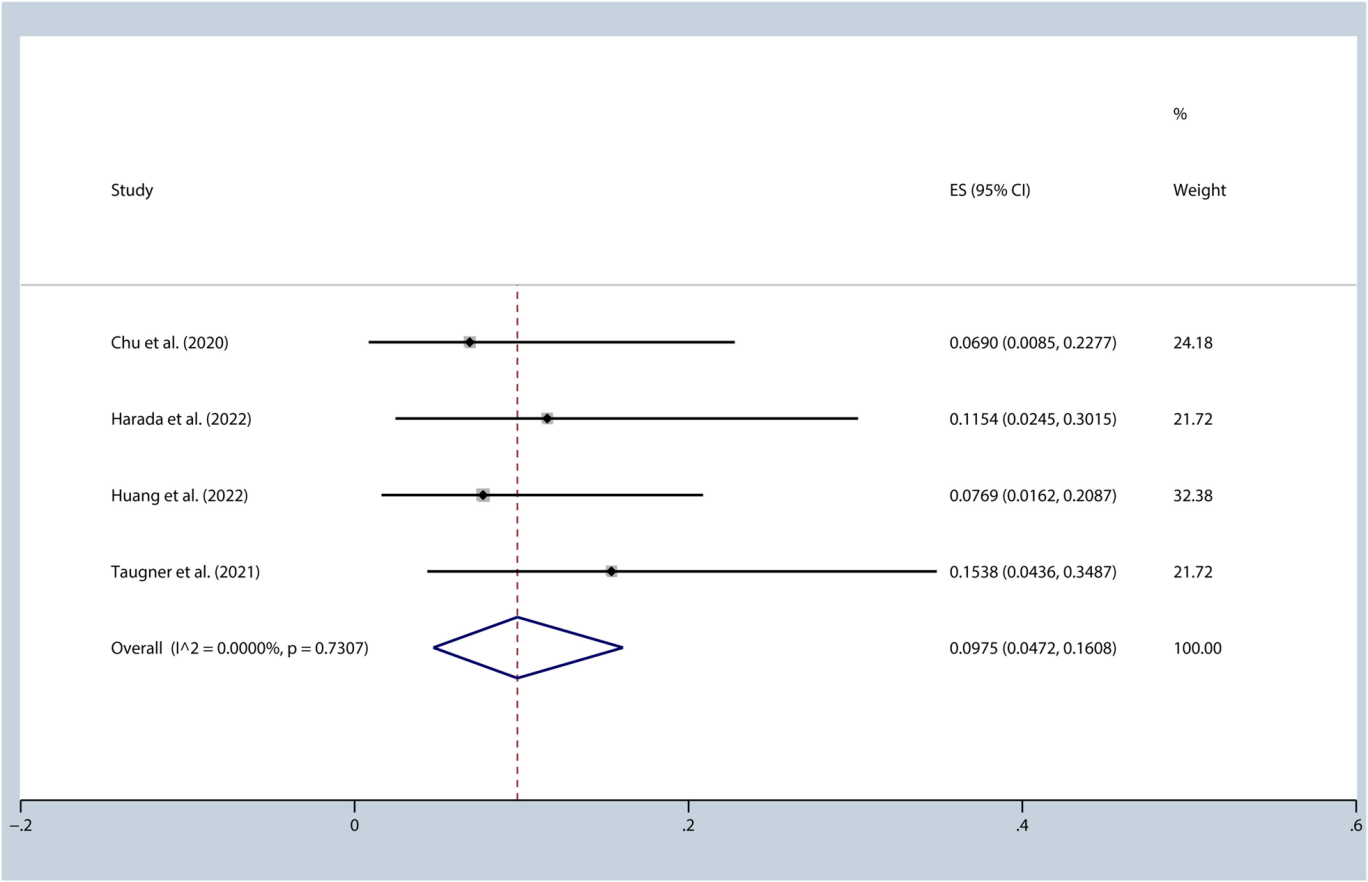
Incidence of pneumonitis (≥grade 3) (radiation dose ≥60Gy)

 All seven studies included in our study had administered a radiotherapy dose of 54 Gy or higher. Furthermore, within these seven studies, we additionally examined a subset of four studies that specifically administered a radiotherapy dose of 60 Gy or higher [29, 31, 32, 35]. This analysis allowed for a comparison between two groups based on radiotherapy dosage, specifically studying the potential effects and outcomes associated with a higher radiotherapy dose (≥ 60 Gy) compared to the overall group of studies that used a dose of ≥ 54 Gy. By conducting this subgroup analysis, we aimed to investigate whether the higher radiotherapy dose had any significant impact on the outcomes of interest. It allows for a more nuanced understanding of the relationship between radiotherapy dose and pneumonitis. The incidence of pneumonitis (Grade ≥ 3) was 9.75% [95%CI (0.05, 0.16), I^2^ = 0.00%, *p* = 0.73] (Fig. 5), and the 1-year PFS rate was 62.05% [95%CI (0.60, 0.65), I^2^ = 0.00%, *p* = 0.68].

**Fig. 6:**
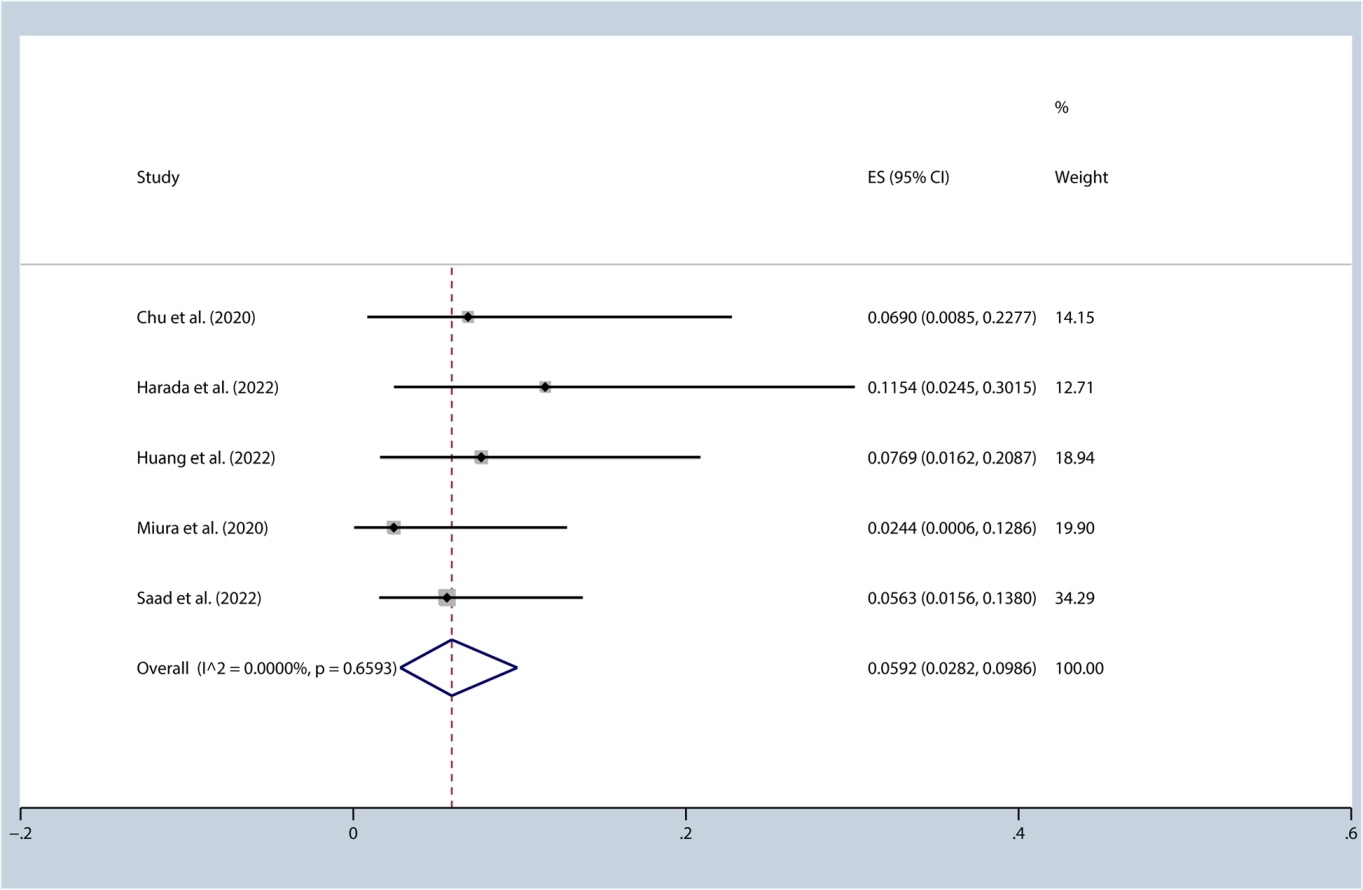
Incidence of pneumonitis (≥grade 3) (Asian)

 We conducted a meta-analysis to investigate how race impacts the risk of pneumonitis (Grade ≥ 3) and analyzed 5 studies that included Asian [29, 31–34]. Our analysis showed a 5.92% [95%CI (0.03, 0.10), I^2^ = 0.00%, *p* = 0.66] incidence of pneumonitis (Grade ≥ 3) (Fig. 6). The I^2^ for the 1-year PFS rate was 74.93%, indicating a high level of heterogeneity. We performed sensitivity analysis and identified that the study conducted by Saad et al. was responsible for the heterogeneity observed [34]. After omitting this study, the I^2^ value reduced significantly to 31.26%, indicative of a substantial decrease in heterogeneity. The 1-year PFS rate was 60.53% [95%CI (0.50, 0.70), I^2^ = 31.26%, *p* = 0.22].

## Discussion

The PACIFIC study showed that using durvalumab treatment 1–42 days after chemoradiotherapy significantly improved overall survival, progression-free survival, and tumor response rates compared to using chemoradiotherapy alone in patients with unresectable locally advanced non-small cell lung cancer [6]. In the latest NCCN guidelines, receiving durvalumab treatment after chemoradiotherapy has become the standard treatment option for these patients, although the guidelines do not specify the optimal duration between the last radiation therapy session and durvalumab treatment. As a result, there is a new focus of research on the safety of using durvalumab treatment between 1 and 42 days after completion of radiation therapy. To evaluate the safety of this treatment regimen, we conducted a meta-analysis that mainly focused on whether the duration between completion of radiotherapy and durvalumab treatment was related to the risk of grade 3 or higher pneumonitis in patients with unresectable locally advanced non-small cell lung cancer.

The results of the meta-analysis showed that the incidence of pneumonitis (Grade ≥ 3) was 5.36% [95%CI (0.03, 0.08), I^2^ = 18.41%, *p* = 0.29], and the 1-year PFS was 57.91% [95%CI (0.53, 0.63), I^2^ = 10.57%, *p* = 0.35]. When the time interval between the completion of radiotherapy and durvalumab treatment was 1–42 days, the incidence of pneumonitis (Grade ≥ 3) was 4.12% [95%CI (0.02, 0.06), I^2^ = 0.00%, *p* = 0.56], and the 1-year PFS was 61.03% [95%CI (0.51, 0.71), I^2^ = 59.06%, *p* = 0.09]. The Chu et al. and Saad et al. studies had intervals longer than 42 days. The incidence of pneumonitis (Grade ≥ 3) and 1-year PFS were 5.63% and 56.40%, respectively, in the Chu et al. study [29], while the incidence of pneumonitis (Grade ≥ 3) was 6.90% in the Saad et al. study [34]. Girard et al. reported that an interval of less than 42 days between the completion of radiotherapy and durvalumab treatment was associated with higher PFS compared to intervals longer than 42 days [30]. Most of the included studies in this analysis were single-arm retrospective studies, making it difficult to compare the results and determine whether there is a statistically significant difference in the incidence of pneumonitis and PFS between the two different time intervals. However, based on these data, it appears that there is no significant increase in pneumonitis or decrease in progression-free survival (PFS) when the time interval is less than 42 days. Subgroup analysis of the PACIFIC trial also suggested that time interval less than 14 days may further improve OS, and there was no difference in the incidence of pneumonitis between patients with time interval less than or greater than 14 days [6]. This finding suggests that a shorter interval between treatment sessions does not appear to have a detrimental effect on these outcomes. Studies have shown that lung tissues from patients with radiation-induced pneumonitis exhibit significant infiltration of lymphocytes [36], while lung tissue and bronchoalveolar lavage fluid from patients with typical immune-related pneumonitis display increased lymphocytes rich in CD8 + T cells [37]. Another similar study found that CD4 + T cells dominate in the bronchoalveolar lavage fluid of patients with immune-related pneumonitis [38]. These findings underscore the cytotoxicity of T cells in inducing radiation-induced and immune-related pneumonitis. Radiation therapy can induce damage to tumor cell DNA and other cellular components, leading to clearance of damaged tumor cells by antigen-presenting cells and increased activation of T cells [39]. Most tumor-specific tumor-infiltrating lymphocytes express T cell receptors that were not identified before immunotherapy, suggesting that these tumor-infiltrating lymphocytes are newly recruited after treatment [40]. Both radiation therapy and immunotherapy recruit lymphocytes, which may be significant causes of radiation-induced and immune-related pneumonitis. Thus, we suggest that patients receive durvalumab treatment within 1–42 days after the completion of radiotherapy, with particular attention to patients receiving durvalumab treatment within 1–14 days after the completion of radiotherapy, and to remain vigilant for possible pneumonitis.

The incidence of pneumonitis may be related to different radiation therapy techniques and plans. Nonetheless, we evaluated the impact of radiation dose on the incidence of grade ≥ 3 pneumonitis and found that among the four studies with a radiation dose of ≥ 60 Gy, the incidence of grade 3 pneumonitis was 9.75% [95%CI (0.05, 0.16), I^2^ = 0.00%, *p* = 0.73]. Additionally, the 1-year progression-free survival (PFS) rate was 62.05% [95%CI (0.60, 0.65), I^2^ = 0.00%, *p* = 0.68].It appears that the incidence of grade ≥ 3 pneumonitis significantly increased in studies with a radiation dose of ≥ 60 Gy. However, subdividing studies based solely on the radiation dose allowed may not be sufficient to accurately assess the impact of the dose on pneumonitis rates. It is important to note that including patients from studies with minimum doses of 54 or 60 Gy does not provide a comprehensive quantification of the actual received dose.Therefore, while our analysis suggests an association between a radiation dose of ≥ 60 Gy and higher incidence of grade ≥ 3 pneumonitis, it is crucial to consider other factors, such as the actual received dose and individual patient characteristics, when assessing the impact of radiation dose on pneumonitis rates. Additionally, new technologies, such as respiratory motion management and image-guided radiation therapy, have the potential to further reduce the risk of pneumonitis. The lung volume receiving a dose of 20 Gy (V20) and the lung volume receiving a dose of 40 Gy (V40) [30, 41] may also be linked to the incidence of pneumonitis. However, due to the lack of relevant data, we cannot further assess the impact of radiation therapy dose, techniques, V20, and V40 on the incidence of pneumonitis. Indeed, conducting further research that includes more precise and comprehensive measurements of radiotherapy would be highly beneficial in gaining a more nuanced understanding of the relationship between radiation therapy and pneumonitis. By capturing detailed data on various aspects of radiotherapy, such as fractionation schedules, treatment techniques, target volumes, and dose distribution, researchers can better assess the impact of these factors on pneumonitis incidence and severity. A more comprehensive approach to data collection and analysis would enable the identification of potential dose-response relationships, the exploration of optimal dose thresholds for minimizing pneumonitis risk, and the development of tailored treatment strategies for patients. Additionally, by considering individual patient characteristics, such as pre-existing lung conditions or genetic susceptibility, future studies can provide a more personalized assessment of the risk associated with radiotherapy-induced pneumonitis. Ultimately, advancing our understanding of the intricacies between radiotherapy parameters and pneumonitis outcomes can contribute to improved treatment planning, individualized patient care, and the optimization of radiotherapy protocols to minimize the risk of pneumonitis while maximizing treatment efficacy. This study analyzed how different races impact the risk of pneumonitis (Grade ≥ 3). Five of the included studies focused on Asian populations, revealing a 5.92% [95%CI (0.03, 0.10), I^2^ = 0.00%, *p* = 0.66] incidence of pneumonitis (Grade ≥ 3) and a 60.53% [95%CI (0.50, 0.70), I^2^ = 31.26%, *p* = 0.22] 1-year PFS rate. This suggests that Asians may have a higher risk of pneumonitis (Grade ≥ 3) but may also benefit more from PFS. However, the study’s conclusion should be approached with caution due to the presence of unaccounted-for influencing factors.

Furthermore, factors such as age, sex, physical fitness score, smoking status, histopathological manifestations, lung dose-volume index, and PD-L1 expression level may also impact the incidence of pneumonitis. For example, the PACIFIC study found that Asian patients with non-squamous tumors and poor physical fitness scores were more likely to develop pneumonitis [5]. Additionally, current smokers may have a lower risk of pneumonitis [42]. Nonetheless, the influence of these factors on the risk of pneumonitis cannot be fully evaluated due to a lack of data.

### Limitations

This study has several limitations: (1) The meta-analysis included seven studies with small sample sizes, which may have influenced the statistical results. (2) Due to the absence of randomized controlled trials with varying time intervals, it is not possible to conduct a direct comparison of the effects of different time intervals on the incidence of pneumonitis. (3) Six studies analyzed were retrospective, which may affect the reliability of the data. (4) Although we intended to conduct a subgroup analysis on aspects such as radiotherapy technology, radiotherapy plan, and PD-L1 status, the current data do not support such analysis.

## Conclusion

Overall, based on the available evidence, it appears that there is no significant increase in pneumonitis or decrease in progression-free survival (PFS) when the time interval is less than 42 days and a shorter interval between treatment sessions does not necessarily have a detrimental effect on the rate of pneumonitis. However, it is important to note that this conclusion is based on the current data and may be subject to limitations and variability among studies. We strongly recommend that clinicians carefully evaluate the specific circumstances of each patient to determine the optimal timing for initiating immunotherapy. Developing an individualized treatment plan that considers various factors such as the patient’s overall health, disease stage, specific cancer type, and treatment goals is crucial. For patients who have started immunotherapy earlier, close monitoring is essential. Regular and thorough observation of these patients can help identify any potential adverse reactions or treatment-related complications, allowing for timely intervention and management.

## Data Availability

The datasets used and/or analyzed during the current study available from the corresponding author on reasonable request.
